# Correlation of neuropsychological and metabolic changes after epilepsy surgery in patients with left mesial temporal lobe epilepsy with hippocampal sclerosis

**DOI:** 10.1186/s13550-018-0385-5

**Published:** 2018-04-12

**Authors:** Canan Güvenç, Patrick Dupont, Jan Van den Stock, Laura Seynaeve, Kathleen Porke, Eva Dries, Karen Van Bouwel, Johannes van Loon, Tom Theys, Karolien E. Goffin, Wim Van Paesschen

**Affiliations:** 1 Department of Neurology, Laboratory for Epilepsy Research, University Hospitals and KU Leuven, Leuven, Belgium; 2 Laboratory for Cognitive Neurology, KU Leuven, Leuven, Belgium; 3 Laboratory for Translational Neuropsychiatry, KU Leuven, Leuven, Belgium; 4 Department of Neurosurgery, University Hospitals and KU Leuven, Leuven, Belgium; 5 Nuclear Medicine and Molecular Imaging, University Hospitals Leuven, Leuven, Belgium; 6 Department of Imaging and Pathology, KU Leuven, Leuven, Belgium

**Keywords:** FDG-PET metabolism, Neuropsychological test scores

## Abstract

**Background:**

Epilepsy surgery often causes changes in cognition and cerebral glucose metabolism. Our aim was to explore relationships between pre- and postoperative cerebral metabolism as measured with ^18^F-fluorodeoxyglucose positron emission tomography (FDG-PET) and neuropsychological test scores in patients with left mesial temporal lobe epilepsy with hippocampal sclerosis (MTLE-HS), who were rendered seizure-free after epilepsy surgery.

**Results:**

Thirteen patients were included. All had neuropsychological testing and an interictal FDG-PET scan of the brain pre- and postoperative. Correlations between changes in neuropsychological test scores and metabolism were examined using statistical parametric mapping (SPM). There were no significant changes in the neuropsychological test scores pre- and postoperatively at the group level. Decreased metabolism was observed in the left mesial temporal regions and occipital lobe. Increased metabolism was observed in the bi-frontal and right parietal lobes, temporal lobes, occipital lobes, thalamus, cerebellum, and vermis. In these regions, we did not find a correlation between changes in metabolism and neuropsychological test scores. A significant negative correlation, however, was found between metabolic changes in the precuneus and Boston Naming Test (BNT) scores.

**Conclusions:**

There are significant metabolic decreases in the left mesial temporal regions and increases in the bi-frontal lobes; right parietal, temporal, and occipital lobes; right thalamus; cerebellum; and vermis in patients with left MTLE-HS who were rendered seizure-free after epilepsy surgery. We could not confirm that these changes translate into significant cognitive changes. A significant negative correlation was found between changes in confrontation naming and changes in metabolism in the precuneus. We speculate that the precuneus may play a compensatory role in patients with postoperative naming difficulties after left TLE surgery. Understanding of these neural mechanisms may aid in designing cognitive rehabilitation strategies.

**Electronic supplementary material:**

The online version of this article (10.1186/s13550-018-0385-5) contains supplementary material, which is available to authorized users.

## Background

Epilepsy is the most common serious neurological disease, with a prevalence of 0.5–1%. Approximately 30% of patients with epilepsy continue to have seizures despite anti-epileptic drug (AED) treatment [1]. Mesial temporal lobe epilepsy due to hippocampal sclerosis (MTLE-HS) is a common form of drug-resistant epilepsy. The resection of mesial temporal structures has been indicated for the treatment of drug-resistant mesial temporal lobe epilepsy (MTLE), with postoperative seizure-free rates of around 65% [2].

Considerable effort has been made to clarify the neurocognitive outcome of patients with MTLE, who undergo epilepsy surgery. Epilepsy surgery for MTLE may lead to cognitive impairments, but also improvements. Deficits in visual motor tasks, mental flexibility, (verbal) memory, reaction times, and attention have been reported in MTLE patients after surgery. In left-sided temporal lobe surgery, there is an increased likelihood of a reduced verbal memory and naming abilities, but improved verbal fluency [3, 4]. A better cognitive outcome has been reported with right-sided hippocampal sclerosis (HS), selective resection of mesial structures, and postoperative seizure freedom [5]. In addition, studies reported a better neuropsychological outcome associated with a younger age at the time of surgery and a shorter duration of TLE [6, 7].

An important tool in the presurgical evaluation of MTLE patients is functional neuroimaging with ^18^F-fluorodeoxyglucose positron emission tomography (FDG-PET). FDG-PET measures regional cerebral glucose metabolism semi-quantitatively and visualizes the distribution of altered glucose metabolism [8, 9]. FDG-PET scans reliably lateralize the seizure focus in patients with MTLE, with decreased metabolism in the epileptogenic temporal lobe [10–12]. Absolute cerebral glucose metabolism may be decreased by 10–30% as a result of AED use [13, 14]. FDG-PET may be a reliable indicator of clinical outcome after surgery as greater severity of preoperative hypometabolism in the resected temporal lobe is associated with significantly better postoperative seizure control [15–17]. Ipsilateral hypometabolism showed a predictive value of 86% for good outcome in a meta-analysis of 46 studies [18]. However, interictal hypometabolism remote to the ictal onset zone is also often noted and is related to a poor surgical outcome [19, 20]. Also, a significant relation between the time of the last seizure and the degree of observed regional hypometabolism in epilepsy patients has been reported [21]. The exact underlying mechanism for the interictal hypometabolism is not fully understood. In this regard, the hypometabolic area seen on interictal FDG-PET is typically larger than the abnormality identified on structural imaging and may extend beyond the temporal lobe, likely representing areas of seizure propagation [22]. Thus, PET images of regional hypometabolism should be interpreted as evidence of a dysfunctional neural network. Language-dominant temporal lobe hypometabolism in MTLE was associated with relatively inferior verbal memory, while nondominant temporal lobe hypometabolism was associated with inferior nonverbal memory [23].

Although many studies have investigated the cognitive outcome of epilepsy surgery, few have focused on the correlation between neuropsychological and metabolic changes in patients with MTLE-HS pre- and postoperative. While some studies find a postoperative (compared to preoperative) increase in cerebral glucose metabolism associated with cognitive impairments, others describe a postoperative decrease in cerebral glucose metabolism associated with cognitive deficits [24–26]. Takaya et al. found that postoperative glucose metabolism was increased compared to the preoperative state in the frontal and parietal lobes as well as in the remaining temporal lobe regions remote from the resected mesial temporal region [27]. Postoperative glucose metabolism was decreased only in the mesial temporal area adjacent to the resected region. Postoperative verbal memory, delayed recall, and attention/concentration scores were significantly better than preoperative scores regardless of the resected side.

We reported previously that the interictal hypometabolism in MTLE-HS was largest in the ipsilateral frontal lobe and represented a seizure-related dynamic process in view of further ictal perfusion decreases [28]. We speculated that surround inhibition in the frontal lobe is a dynamic defense mechanism against seizure propagation and may be responsible for functional deficits observed in MTLE. We postulated that epilepsy surgery can also be seen as a release of the brakes on the surrounding cortex. The aim of our study, therefore, was to investigate the relationships between interictal cerebral glucose metabolism as measured by FDG-PET and neuropsychological performance in seizure-free MTLE-HS patients pre- and postoperative. We hypothesized that cognitive changes would correlate with metabolic changes.

## Methods

### Subjects

We retrospectively selected patients who met the following inclusion criteria: (1) refractory left MTLE-HS, (2) preoperative evaluation and epilepsy surgery, (3) seizure freedom for at least 1 year after epilepsy surgery, and (4) pre- and postoperative neuropsychological assessment and imaging as described below. Informed consent was obtained from all participants before the investigations. The study was approved by the institutional review board of the university hospitals UZ Leuven-KU Leuven.

### Neuropsychological assessment

Patients underwent comprehensive preoperative and postoperative neuropsychologic assessment including Digit Span subtest of Wechsler Memory Scale-Revised, Rey Auditory Verbal Learning Test (RAVLT), Rey Visual Design Learning Test (RVDLT), verbal fluency (Controlled Oral Word Association Test), response-inhibition (Stroop Color-Word, Stroop IF Test) and set-shifting (Trail Making Test), and Boston naming test (BNT) [29, 30]. *z*-scores were derived by comparing the test results to a normal control population.

### Imaging protocol and processing

An FDG-PET scan was acquired 30 min after injection of 150 MBq ^18^F-fluorodeoxyglucose (FDG) under continuous EEG monitoring, using an ECAT EXACT HR+ PET scanner. Prior to the injection, a transmission scan was performed. FDG-PET data were acquired as six frames of 5 min and reconstructed using an iterative reconstruction algorithm including corrections for randoms, dead time, scatter, and attenuation, and taking into account the finite point spread function of the PET system [31, 32]. Images were reconstructed using ordered subsets expectation-maximization (OSEM) algorithm with the number of iterations and subsets equivalent to 250 MLEM iterations. A correction for small movements was applied using the realignment function in SPM12 (Wellcome Department of Cognitive Neurology, London, UK), and images were summed. These images represent the tracer distribution of FDG in the brain between 30- and 60-min postinjection.

All subjects underwent high-resolution MR imaging (T1- and T2-weighted sequences and a magnetization-prepared rapid gradient echo (MPRAGE)), which was performed on a 3T Vision Scanner (Siemens, Erlangen, Germany).

For each patient, the preoperative FDG-PET image was co-registered to the preoperative structural MRI (MPRAGE) using SPM12, and the postoperative FDG-PET image was co-registered to the postoperative structural MRI (MPRAGE). Next, we performed a rigid body co-registration of the postoperative to the preoperative MRI scans followed by high-resolution warping of the pre- and postoperative structural MRI scans to account for the shift in anatomical structures in the brain due to the surgery. This image transformation was then applied to the corresponding FDG-PET images so that the deformation between preoperative and postoperative scans was taken into account. The preoperative MRI was segmented into gray matter (GM), white matter (WM), and cerebrospinal fluid (CSF) using SPM12. During this segmentation step, we also obtained the warping to the stereotactic Montreal Neurological Institute (MNI) space, and this transformation was applied to all MRI and PET data of that patient. Finally, the warped PET images were smoothed with an isotropic Gaussian 3D kernel with a full width at half maximum (FWHM) of 12 mm. Because we expected changes of metabolism in gray matter, we applied a gray matter mask. This mask was constructed based on all individual postoperative GM masks defined by taking all voxels with GM > 0.5. Since we were interested in the regional changes, we normalized the images by dividing them by the total activity within the final GM mask. Note that this procedure leads to a unit-less value in which differences in administered tracer activity or blood glucose levels between pre- and postoperative conditions or between patients are canceled out. This corrected image is a surrogate measure for glucose metabolism in the brain and reflects the regional distribution of glucose metabolism. Changes in the distribution of regional cerebral glucose metabolism were calculated as a difference image between the corrected postoperative and preoperative FDG-PET images.

### Statistical analysis

Neuropsychological data analysis was performed using SPSS statistical software (IBM SPSS Statistics 20; Chicago, IL, USA). A paired *t* test was used to determine whether there was a significant difference in the neuropsychological test score post- versus preoperatively (*p* < 0.05). Changes in neuropsychological test scores were calculated as the postoperative value minus the preoperative value.

A voxel-based paired *t* test was used to identify voxels which differ significantly in preoperative versus postoperative regional distribution of FDG metabolism. A voxel-based regression analysis was performed to identify changes in FDG metabolism related to the changes in cognitive functioning. For all voxel-based analyses, we used an uncorrected *p* < 0.001 at the voxel level combined with a FWE-corrected *p* < 0.05 at the cluster level.

## Results

### Patients

Thirteen patients with drug-resistant left MTLE-HS, who underwent epilepsy surgery and were rendered seizure-free, were included. Table 1 gives an overview of the demographic variables. The median time interval between pre- and postoperative neuropsychological testing was 937 days (range 525–2192 days) and between pre- and postoperative PET acquisition 816 days (range 518–2293 days), respectively. Neuropsychological testing and FDG-PET were performed at a median of 140 days (range 35–239 days) and 148 days (range 66–330 days) prior to surgery, respectively, and at a median of 847 days (range 375–2107 days) and 673 days (range 370–2200 days) after surgery (*p* > 0.05), respectively. Patients were taking AEDs at the time of preoperative FDG-PET and neuropsychological testing. As a result of change or elimination of one of the AEDs postoperative, nine patients had a different AED schedule at the time of the postoperative imaging and neuropsychological testing in comparison with preoperatively (Additional file 1: Table S1). Eleven patients were strictly right-handed. Two patients were left-handed, but the language was left lateralized; one subject underwent a language fMRI and had a left lateralization, and the second subject had postictal aphasia with clear left unilateral seizures, which is a strong indication for left lateralized language. A standard temporal lobe resection, including the hippocampus, was performed in all 13 patients with left MTLE-HS.

**Table 1:** Demographic variables of patients with left MTLE-HS

Characteristics	*n* (%)	Median	Range
Total population	13 (100%)		
Age (years)		47	17–59
Education (years)		12	10–18
Age at onset (years)		8	0–37
Age at surgery (years)		44	16–59
Duration of epilepsy (years)		32	8–51
Handedness			
Left	2 (15%)		
Right	11 (85%)		
Gender			
Female	6 (46%)		
Male	7 (54%)		

*n* number

### Changes in neuropsychological measures after epilepsy surgery

Neuropsychological test scores and *z*-scores before and after epilepsy surgery are summarized in Table 2. No significant differences at the group level were found between the pre- and postoperative neuropsychological test scores.

**Table 2:** Changes in neuropsychological measures after epilepsy surgery

Neuropsychological test	Preoperative		Postoperative		*p* value
	*z*-score	Test score	*z*-score	Test score	
RAVLT					
Sum (A1–A5)	− 1.13 ± 1.76	36.85 ± 9.79	− 1.88 ± 1.16	34.00 ± 10.07	0.31
Delayed recall	− 2.43 ± 1.04	4.92 ± 2.47	− 2.11 ± 0.61	4.54 ± 1.85	0.53
Recognition (+)	− 1.40 ± 1.65	12.38 ± 2.57	− 1.63 ± 2.13	11.77 ± 2.80	0.35
RVDLT					
Sum (A1–A5)	− 0.88 ± 1.16	28.75 ± 10.96	− 1.30 ± 1.38	28.67 ± 12.29	0.96
Delayed recall	− 1.82 ± 2.01	5.44 ± 2.06	− 1.23 ± 1.49	6.56 ± 3.47	0.53
Recognition (+)	− 0.34 ± 1.14	12.17 ± 1.64	− 0.94 ± 1.81	12.67 ± 2.10	0.39
Stroop Test	− 3.24 ± 2.85	28.00 ± 31.96	− 2.89 ± 3.09	37.77 ± 34.09	0.18
Trail Making Test	0.93 ± 0.33	81.77 ± 8.49	1.15 ± 0.54	84.54 ± 11.53	0.27
Verbal fluency					
Letter fluency (NAK)	− 1.39 ± 3.89	22.08 ± 10.77	− 0.51 ± 4.61	24.46 ± 12.82	0.29
AVF (animal verbal fluency)	− 1.96 ± 0.82	13.23 ± 4.15	− 1.74 ± 0.78	14.92 ± 4.37	0.20
Boston Naming Test	− 3.88 ± 1.88	41.46 ± 6.59	− 4.07 ± 2.17	40.85 ± 8.17	0.70
Digit Span					
Forward	− 0.98 ± 1.19	5.00 ± 1.05	− 0.27 ± 1.23	5.50 ± 0.97	0.14
Backward	− 1.68 ± 1.54	4.00 ± 1.41	− 2.10 ± 0.96	3.80 ± 0.92	0.56

Data are presented as mean ± standard deviation

Stroop Test—an interference measure was calculated by subtracting the average time needed to complete the first two subtasks from the time needed to complete the third subtask (Interference = Stroop III − [(Stroop I + Stroop II)/2])

Trail Making Test—based on the direct time scores, derived scores were calculated as ratio score (B/A). *p* value is for a paired *t* test

*RAVLT* Rey Auditory Verbal Learning Test, *RVDLT* Rey Visual Design Learning Test

### Changes in FDG-PET metabolism after epilepsy surgery

Visual assessment of baseline preoperative FDG-PET scans showed that all patients had left temporal lobe hypometabolism, four (31%) had in addition right temporal lobe hypometabolism and five patients (38%) had also hypometabolism elsewhere in the brain.

**Fig. 1:**
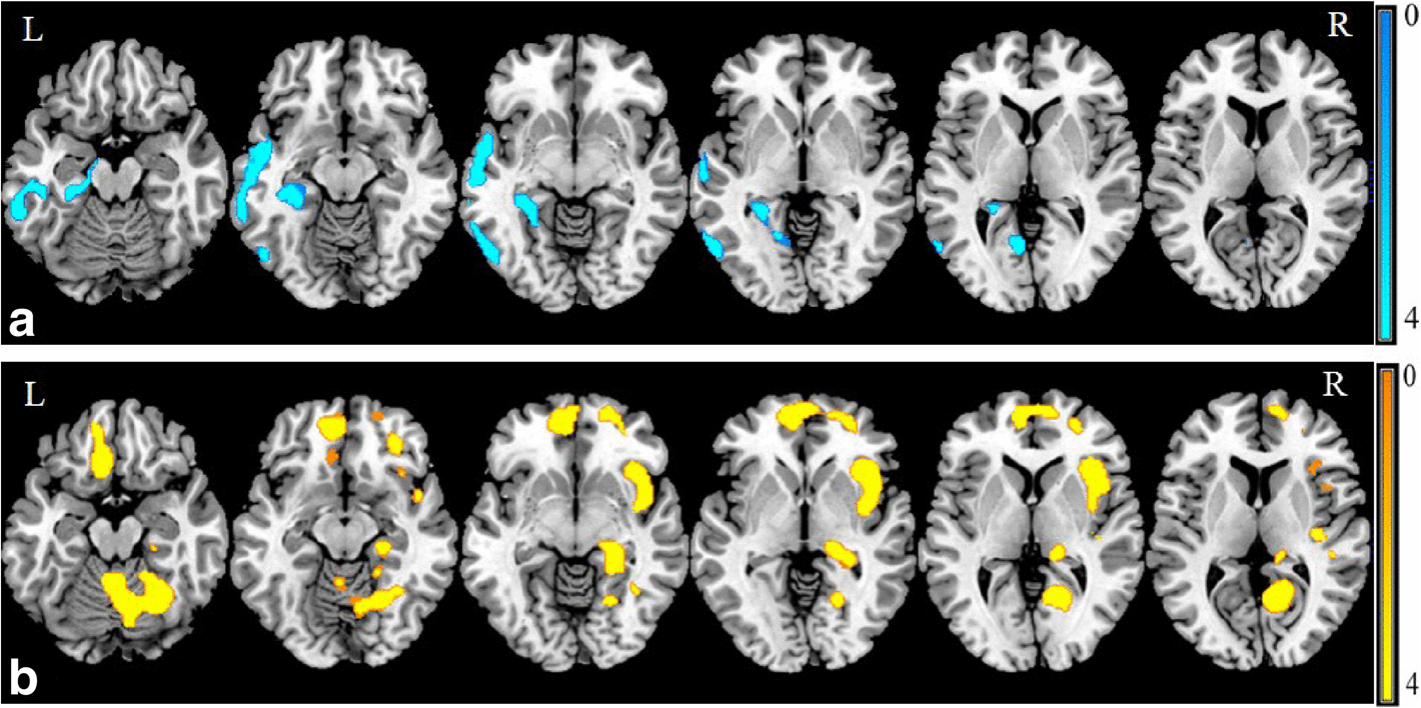
SPMt map of significant changes in brain metabolism after epilepsy surgery. **a** Regions with significant decreased metabolism (blue) were mainly in the left temporal and occipital lobes. **b** Regions with significant increased metabolism (yellow) were mainly in the bi-frontal, right temporal, occipital, and parietal lobes; right thalamus; cerebellum; and vermis. SPMt maps were thresholded at uncorrected *p* < 0.001 at the voxel level combined with a FWE-corrected *p* < 0.05 at the cluster level

A voxel-based comparison of postoperative metabolism compared to preoperative metabolism showed decreased metabolism in the left temporal and occipital lobes (Fig. 1, Additional file 2: Table S2). Increased metabolism was present in the bi-frontal lobes; right parietal, temporal, and occipital lobes; right thalamus; cerebellum; and vermis (Fig. 1, Additional file 2: Table S2). In more detail, metabolism was decreased postoperatively in the left mesial temporal region, more specifically in the superior, inferior, and middle temporal gyrus, and fusiform gyrus extending to the left hippocampus and lingual gyrus (Fig. 1a). Metabolism was increased postoperatively in the right insula and left gyrus rectus extending to inferior, superior, and middle frontal gyrus, supplementary motor area, right pre- and postcentral gyrus, right hippocampus, right lingual gyrus, right fusiform gyrus, right thalamus, cerebellum, and vermis (Fig. 1b).

### Correlations between neuropsychological and metabolic changes after epilepsy surgery

**Fig. 2:**
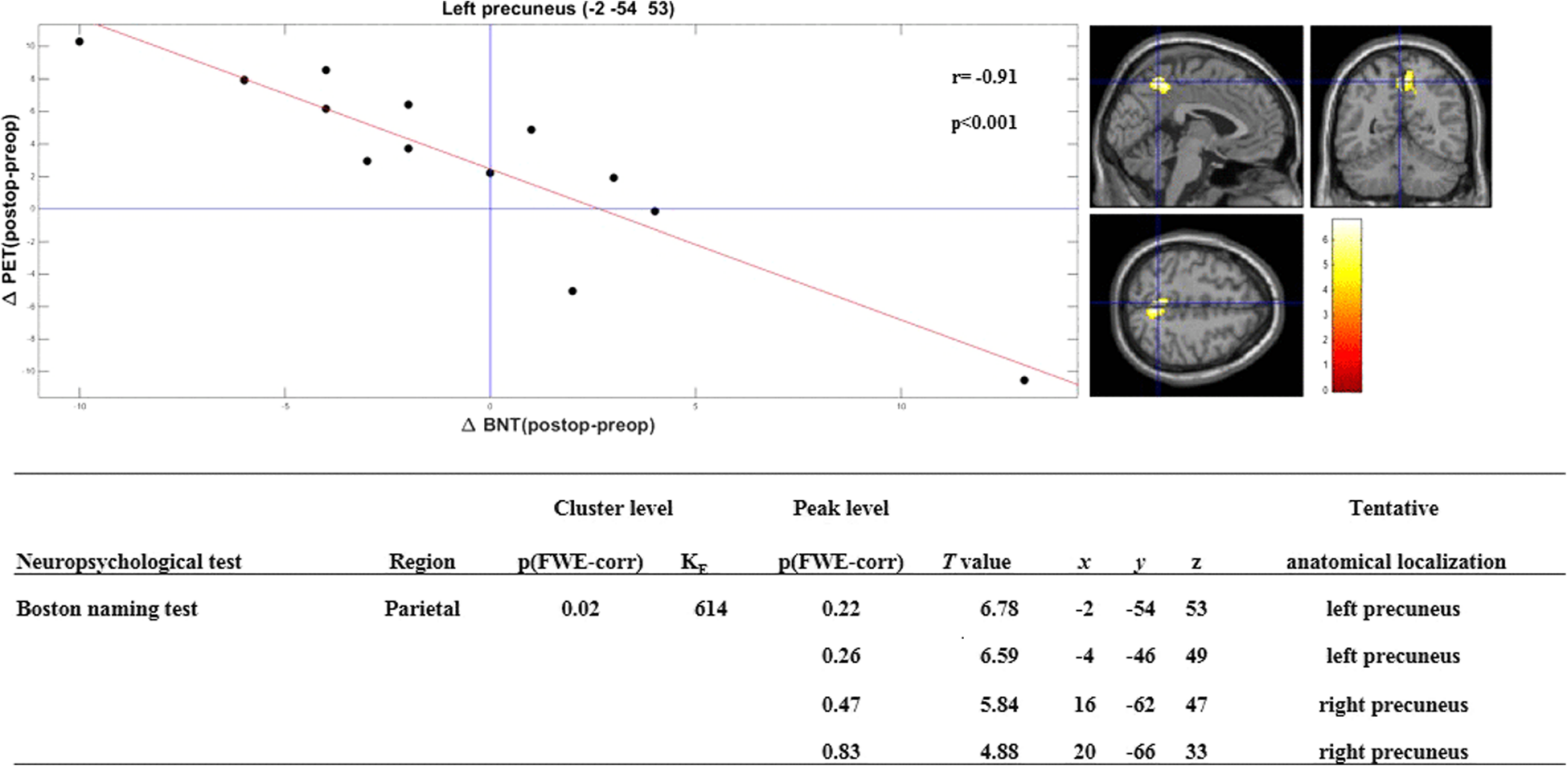
Negative correlation between Boston Naming Test (BNT) and metabolic changes after epilepsy surgery in the precuneus. The blue cross is positioned in the left precuneus (*x*, − 2; *y*, − 54; *z*, 53). Changes in metabolism and neuropsychological test were calculated as the postoperative values minus the preoperative corrected FDG-PET images. SPMt maps were thresholded at uncorrected *p* < 0.001 at the voxel level combined with a FWE-corrected *p* < 0.05 at the cluster level

In the brain regions with significant metabolic changes, we did not find a correlation between changes in metabolism and Digit Span subtest of Wechsler Memory Scale-Revised, RAVLT, RVDLT, verbal fluency, response-inhibition and set-shifting (Trail Making Test), and BNT. A significant negative correlation was found between the changes in metabolism and changes in BNT scores postoperatively compared to preoperatively in the precuneus bilaterally (*r* = − 0.91, *p* < 0.001) (Fig. 2).

## Discussion

The primary aim of our study was to explore whether there exists a relationship between changes in interictal FDG-PET cerebral glucose metabolism and neuropsychological test scores after epilepsy surgery in seizure-free left MTLE-HS patients. Our study showed on one hand a lack of correlation between interictal cerebral glucose metabolism and neuropsychological performance, and on the other hand, a negative correlation between changes in BNT and metabolism in the precuneus.

Patients with left MTLE-HS are at risk of having a decrease in verbal memory and naming abilities but may experience an improvement in verbal fluency [3, 5]. Although our patients as a group scored lower on verbal memory tests and BNT, and better on verbal fluency tasks after surgery, the results were not significant. FDG-PET metabolism after epilepsy surgery decreased in the left temporal and occipital lobes and increased in the bi-frontal lobes; right parietal, temporal, and occipital lobes; right thalamus; cerebellum; and vermis, which confirms previous reports [23, 27]. Suggested mechanisms underlying increased postoperative brain metabolism include the recovery of impaired brain activity after the cessation of interictal discharges by surgery. The recovery of metabolic function could be seen as a reflection of the plasticity of anatomical connections [33]. It is possible that the recovery of metabolic function in these regions can result in the rearrangement and, possibly, an increase in the number of connections from the remaining parts of the brain and, as a result, in a recovery of brain activity. We, however, found no correlations between changes in metabolism and Digit Span subtest of Wechsler Memory Scale-Revised, RAVLT, RVDLT, verbal fluency, response-inhibition and set-shifting (Trail Making Test), and BNT after epilepsy surgery in seizure-free left MTLE-HS patients. Unexpectedly, we found a significant negative correlation between changes in BNT and metabolism in the precuneus, i.e., patients with more severe naming difficulties postsurgery had higher metabolism in the precuneus, which has not been reported in the epilepsy literature. Naming declines are common following the left temporal lobe resection and are associated with higher age at seizure onset, higher age at the time of surgery, and better preoperative naming ability [4]. Recently, Zanão and colleagues studied the relationship between the default mode network (DMN) and neuropsychological performance in TLE patients and controls. They reported a disruption of the normal pattern of DMN in TLE, with reduction of temporal lobe connectivity, which could explain a worse performance in verbal memory compared with controls [34].

In patients with left TLE, Bonelli and colleagues demonstrated that left middle and inferior frontal gyri activation during an fMRI verbal fluency task was significantly correlated with naming abilities, most likely reflecting a compensatory response due to the ongoing epileptic activity and/or underlying pathology [35].

In an fMRI study of the effects of attempted naming on word retrieval in two patients with anomic aphasia, picture naming performance improved after multiple presentations over a period of days. At the neural level, the left precuneus in one subject with anomic aphasia and also control subjects was implicated [36–38]. We speculate that the increased metabolism in the precuneus in our patients may represent a compensatory mechanism for the naming difficulties, which warrants further study. Although naming difficulties occur in around 34% of left TLE surgeries, cognitive rehabilitation strategies for naming difficulties have not been established. The strategy of “errorless learning,” in which maximum cues are provided during training so that patients never make mistakes, was successful in one patient [39]. A better understanding of the correlations between cognitive and cerebral metabolic changes after epilepsy surgery could lead to the design of better cognitive rehabilitation strategies. Further investigations with larger sample size are required to confirm the correlation between changes in metabolism and neuropsychological test scores.

## Conclusions

After epilepsy surgery for left mesial temporal lobe epilepsy with hippocampal sclerosis, decreased FDG-PET metabolism was observed in the left mesial temporal regions and increases in the bi-frontal lobes; right parietal, temporal, and occipital lobes; right thalamus; cerebellum; and vermis. We could not confirm that these changes translate into significant cognitive changes. A negative correlation was found between changes in confrontation naming and changes in metabolism in the precuneus. We speculate that the precuneus may play a compensatory role in patients with postoperative naming difficulties after left TLE surgery. Understanding of these neural mechanisms may aid in designing cognitive rehabilitation strategies.

## Additional files

Additional file 1: Anti-epileptic drug treatment preoperative and postoperative. (DOCX 17 kb)

Additional file 2: Changes in brain metabolism between postoperative and preoperative PET. (DOCX 20 kb)
