# Supplementary material for: Correlation of neuropsychological and metabolic changes after epilepsy surgery in patients with left mesial temporal lobe epilepsy with hippocampal sclerosis
Source: EJNMMI Res. 2018 Apr 12;8:31. doi: 10.1186/s13550-018-0385-5 (PMC5897268; doi:10.1186/s13550-018-0385-5)
Supplement: Supplementary file 1 — Anti-epileptic drug treatment preoperative and postoperative. (DOCX 17 kb) [file 13550_2018_385_MOESM1_ESM.docx]

Table S1

Anti-epileptic drug treatment preoperative and postoperative

|  | |  | | | | |  |  | | | | |  | | |  |  |  |  |
| --- | --- | --- | --- | --- | --- | --- | --- | --- | --- | --- | --- | --- | --- | --- | --- | --- | --- | --- | --- |
|  | | **Preoperative** | | | | **Postoperative** | | | | | |  | | |  |  |  |  |  |
| subject | | AED | | Dosis(mg) | | AED | | | | | **Dosis(mg)** | Dosis ∆ | | |  |  |  |  |  |
|  | |  | |  | |  | | |  | | |  | | |  |  |  |  |  |
| S01 | | Carbamazepine | | 1200 | | Carbamazepine | | | 800 | | | -400 | | |  |  |  |  |  |
|  | | Primidon | | 1000 | | Primidon | | | 250 | | | -750 | | |  |  |  |  |  |
|  | | Topiramaat | | 200 | | Topiramaat | | | 50 | | | -150 | | |  |  |  |  |  |
|  | |  | |  | |  | | |  | | |  | | |  |  |  |  |  |
| S02 | | Valproïnezuur | | 1000 | | / | | |  | | |  | | |  |  |  |  |  |
|  | | Lamotrigine | | 200 | | Lamotrigine | | | 150 | | | -50 | | |  |  |  |  |  |
|  | | Tiagabine | | 30 | | / | | |  | | |  | | |  |  |  |  |  |
|  | |  | |  | |  | | |  | | |  | | |  |  |  |  |  |
| S03 | | Carbamazepine | | 1300 | | Carbamazepine | | | 400 | | | -900 | | |  |  |  |  |  |
|  | | Fenobarbital | | 170 | | / | | |  | | |  | | |  |  |  |  |  |
|  | |  | |  | | Tegretol | | | 400 | | |  | | |  |  |  |  |  |
|  | |  | |  | |  | | |  | | |  | | |  |  |  |  |  |
| S04 | | Carbamazepine | | 800 | | Carbamazepine | | | 1200 | | | 400 | | |  |  |  |  |  |
|  | |  | |  | |  | | |  | | |  | | |  |  |  |  |  |
| S05 | | Carbamazepine | | 1400 | | Levetiracetam | | | 1000 | | | -400 | | |  |  |  |  |  |
|  | | Lamotrigine | | 400 | |  | | |  | | |  | | |  |  |  |  |  |
|  | |  | |  | |  | | |  | | |  | | |  |  |  |  |  |
| S06 | | Carbamazepine | | 1400 | | Carbamazepine | | | 1000 | | | -400 | | |  |  |  |  |  |
|  | | Topiramaat | | 200 | | Topiramaat | | | 1450 | | | 1250 | | |  |  |  |  |  |
|  | |  | |  | |  | | |  | | |  | | |  |  |  |  |  |
| S07 | | Carbamazepine | | 400 | | Levetiracetam | | | 400 | | | 0 | | |  |  |  |  |  |
|  | | Topiramaat | | 300 | |  | | |  | | |  | | |  |  |  |  |  |
|  | |  | |  | |  | | |  | | |  | | |  |  |  |  |  |
| S08 | | Lamotrigine | | 300 | | Lamotrigine | | | 30 | | | -270 | | |  |  |  |  |  |
|  | | Topiramaat | | 200 | | Topiramaat | | | 300 | | | 100 | | |  |  |  |  |  |
|  | | Fenobarbital | | 100 | | / | | |  | | |  | | |  |  |  |  |  |
|  | |  | |  | |  | | |  | | |  | | |  |  |  |  |  |
|  | |  | |  | |  | | |  | | |  | | |  |  |  |  |  |
| S09 | | Gabapentine | | 3600 | | Gabapentine | | | 1200 | | | -2400 | | |  |  |  |  |  |
|  | | Lamotrigine | | 250 | | Lamotrigine | | | 250 | | | 0 | | |  |  |  |  |  |
|  | | Levetiracetam | | 3000 | | Levetiracetam | | | 2000 | | | -1000 | | |  |  |  |  |  |
|  | | Vigabatrine | | 2000 | |  | | |  | | | -2000 | | |  |  |  |  |  |
|  | |  | |  | |  | | |  | | |  | | |  |  |  |  |  |
| S10 | | Fenytoine | | 300 | | Fenytoine | | | 400 | | | 100 | | |  |  |  |  |  |
|  | | Levetiracetam | | 500 | | / | | |  | | |  | | |  |  |  |  |  |
|  | | Fenobarbital | | 500 | | Fenobarbital | | | 100 | | | -400 | | |  |  |  |  |  |
|  | |  | |  | |  | | |  | | |  | | |  |  |  |  |  |
| S11 | | Levetiracetam | | 3000 | | Levetiracetam | | | 1500 | | | -1500 | | |  |  |  |  |  |
|  | | Lamotrigine | | 100 | | Lamotrigine | | | 100 | | | 0 | | |  |  |  |  |  |
|  | |  | |  | |  | | |  | | |  | | |  |  |  |  |  |
| S12 | | Levetiracetam | | 1000 | | Levetiracetam | | | 1000 | | | 0 | | |  |  |  |  |  |
|  | | Carbamazepine | | 800 | | Carbamazepine | | | 800 | | | 0 | | |  |  |  |  |  |
|  | |  | |  | |  | | |  | | |  | | |  |  |  |  |  |
| S13 | | Carbamazepine | | 1000 | | Carbamazepine | | | 1000 | | | 0 | | |  |  |  |  |  |
|  | | Levetiracetam | | 200 | | Levetiracetam | | | 200 | | | 0 | | |  |  |  |  |  |
|  | | combi preparaat fenobarbital + fenytoine | | 100 | | combi preparaat fenobarbital + fenytoine | | | 100 | | | 0 | | |  |  |  |  |  |
|  | |  | | | | |  |  | | | | | | | | | |  |  |

Dosis ∆: postoperative dosis (mg) - preoperative dosis (mg). AED: anti-epileptic drug
