# Supplementary material for: Correlation of neuropsychological and metabolic changes after epilepsy surgery in patients with left mesial temporal lobe epilepsy with hippocampal sclerosis
Source: EJNMMI Res. 2018 Apr 12;8:31. doi: 10.1186/s13550-018-0385-5 (PMC5897268; doi:10.1186/s13550-018-0385-5)
Supplement: Supplementary file 2 — Changes in brain metabolism between postoperative and preoperative PET. (DOCX 20 kb) [file 13550_2018_385_MOESM2_ESM.docx]

Table S2

Changes in brain metabolism between postoperative and preoperative PET

|  | Cluster level | | Peak level | |  |  |  |  |  |
| --- | --- | --- | --- | --- | --- | --- | --- | --- | --- |
| Changes brain metabolism | p(FWE-corr) | Cluster size (voxels) | p(FWE-corr) | *T* value | *x* | *y* | *z* |  | Tentative anatomical localization |
|  |  |  |  |  |  |  |  |  |  |
| Decreased | < 0.001 | 1540 | 0.005 | 11.067 | -54 | -2 | -13 |  | Left superior temporal gyrus |
|  |  |  | 0.179 | 6.791 | -58 | -12 | -13 |  | Left middle temporal gyrus |
|  |  |  | 0.278 | 6.303 | -66 | -36 | -15 |  | Left inferior temporal gyrus |
|  |  |  | 0.34 | 6.067 | -26 | -28 | -25 |  | Left fusiform gyrus |
|  |  |  | 0.453 | 5.725 | -32 | -36 | -5 |  | left hippocampus |
|  |  |  | 0.697 | 5.101 | -6 | -62 | 3 |  | Left lingual gyrus |
|  |  |  |  |  |  |  |  |  |  |
| Increased | 0.003 | 852 | 0.011 | 10.004 | 42 | 20 | -3 |  | Right insula |
|  | < 0.001 | 1725 | 0.101 | 7.422 | -8 | 22 | -21 |  | Left rectus |
|  |  |  | 0.186 | 6.753 | -8 | 56 | -3 |  | Left frontal gyrus. medial orbital |
|  |  |  | 0.358 | 6.011 | 10 | 60 | 5 |  | Right superior frontal gyrus. medial |
|  |  |  | 0.728 | 5.024 | 28 | 50 | 3 |  | Right middle frontal gyrus |
|  |  |  | 0.804 | 4.822 | 34 | 38 | -11 |  | Right inferior frontal gyrus. orbital |
|  | < 0.001 | 2898 | 0.145 | 7.022 | -20 | 26 | 51 |  | Left frontal gyrus. medial |
|  |  |  | 0.232 | 6.508 | 0 | 32 | 39 |  | Left superior frontal gyrus. medial |
|  |  |  | 0.284 | 6.281 | 6 | 40 | 43 |  | Right superior frontal gyrus. medial |
|  |  |  | 0.312 | 6.175 | 6 | 8 | 63 |  | Right supplementary motor area |
|  |  |  | 0.415 | 5.835 | -6 | 18 | 53 |  | Left supplementary motor area |
|  |  |  | 0.555 | 5.455 | 46 | 10 | 31 |  | Right precentral gyrus |
|  | 0.035 | 455 | 0.476 | 5.661 | 44 | -16 | 39 |  | Right postcentral gyrus |
|  |  |  | 0.942 | 4.328 | 46 | -32 | 7 |  | Right superior temporal gyrus |
|  | < 0.001 | 3939 | 0.312 | 6.175 | 26 | -32 | -5 |  | Right hippocampus |
|  | < 0.001 | 3939 | 0.103 | 7.394 | 14 | -56 | 7 |  | Right lingual gyrus |
|  |  |  | 0.148 | 7.000 | 24 | -60 | -15 |  | Right fusiform gyrus |
|  |  |  | 0.812 | 4.801 | 16 | -30 | 3 |  | Right thalamus |
|  |  |  | 0.377 | 5.951 | 8 | -62 | -19 |  | Right cerebellum |
|  |  |  | 0.569 | 5.417 | -2 | -52 | -29 |  | Vermis |
|  |  |  | 0.898 | 4.521 | -10 | -74 | -29 |  | Left cerebellum crus |

SPM analysis shows the local maxima after thresholding at P < 0.001 (uncorrected) at voxel level and a FWE-corrected p < 0.05 at the cluster level. x, y, z coordinates in mm in MNI space with respect to the anterior commissure. Abbreviation: FWE-corr: family wise error correction.
